# Supplementary material for: Breast cancer screening practices among Vietnamese women and factors associated with clinical breast examination uptake
Source: PLoS One. 2022 May 27;17(5):e0269228. doi: 10.1371/journal.pone.0269228 (PMC9140272; doi:10.1371/journal.pone.0269228)
Supplement: S1 File — (PDF) [file pone.0269228.s002.pdf]

S1 Table. Sample size calculation of the study

Following the guidelines from WHO for sample size determination in health studies, the sample size was calculated using the formula to estimate a population proportion with specific absolute precision[1].

$$n = de\frac{Z_{1-\alpha/2}^{2}p(1-p)}{d^{2}} + c$$

In which:

p	0.50	The estimated proportion of women who had ever had screening for breast cancer was assumed at 50% to generate the most conservative, or largest, sample size.
$Z_{1-\alpha/2}$	1.96	$Z_{1-\alpha/2}$ has been set at 1.96 which is the corresponding value at 95% level of confidence for a two-tailed test.
d	0.1	The absolute precision was chosen at 0.1 to estimate the prevalence to be within 10 percentage points of the true value.
de	2	The design effect is estimated as 2 to account for the use of cluster sampling (clustering at administrative unit).
c		Contingency = n x non-response rate. A non-response rate of 10% has been accounted for the sample size calculation.
n	211	

Using the formula and above assumptions, the sample size needed was estimated to be 211 which was rounded up to 250. In order to get estimations that would be comparable for urban and rural, the sample size was doubled to 500 of which 250 respondents from the urban areas and 250 respondents from the rural areas.

[1] Lwanga SK, Lemeshow S, World Health O. Sample size determination in health studies : a practical manual / S. K. Lwanga and S. Lemeshow. Geneva: World Health Organization; 1991.

S2 Table. Sample quota by age group

Age group (years old)	Population*	Percentage	Sample size		
			Per commune	Per district	Total
30-39	6,680,741	34.6	43	86	173
40-49	5,844,636	30.2	38	76	151
50-59	3,972,674	20.5	26	51	103
60-74	2,838,356	14.7	18	37	73
**Total**	**19,336,407**	**100**	**125**	**250**	**500**

* *Vietnamese female population by age group reported in Census 2009*[2]

[2] Vietnam General Statistics Office, Central Population and Housing Census Steering Committee. Vietnam Population and Housing Census 2009. Hanoi, Vietnam: Statistical Publishing House; 2010. 882 p.

S1 Fig. Proportion of respondents who could correctly identify symptoms of breast cancer, by residence area.

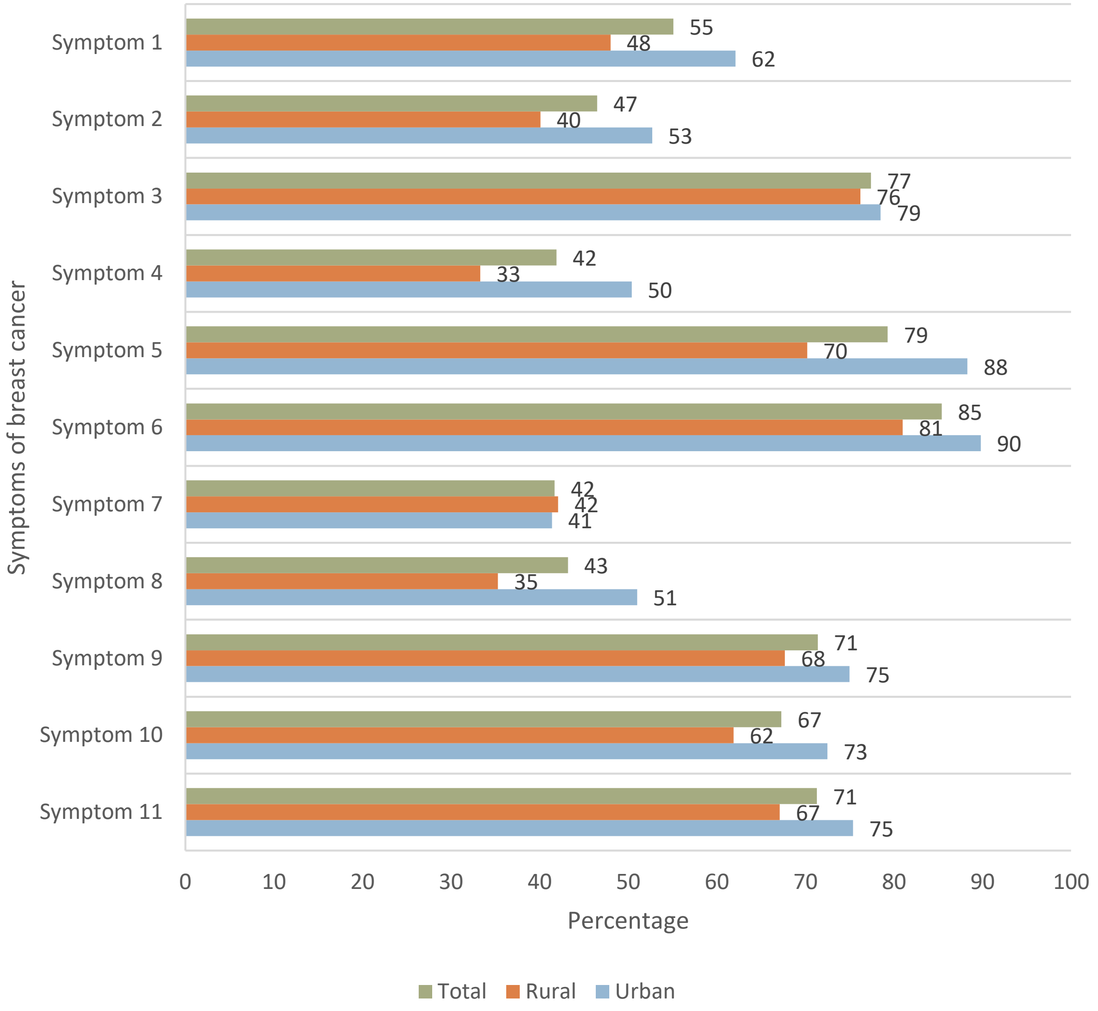

Symptom 1:	Change in nipple position	Symptom 7:	Nipple rash
Symptom 2:	Pulling in of nipple	Symptom 8:	Redness of breast skin
Symptom 3:	Pain in breast/armpit	**Symptom 9:**	**Lump under armpit**
Symptom 4:	Puckering/dimpling of breast skin	Symptom 10:	Change in size of breast/nipple
Symptom 5:	Discharge from nipple	Symptom 11:	Change in shape of breast/nipple
**Symptom 6:**	**Lump in breast**		

S2 Fig. Proportion of respondents who could correctly identify risk factors of breast cancer, by residence area.

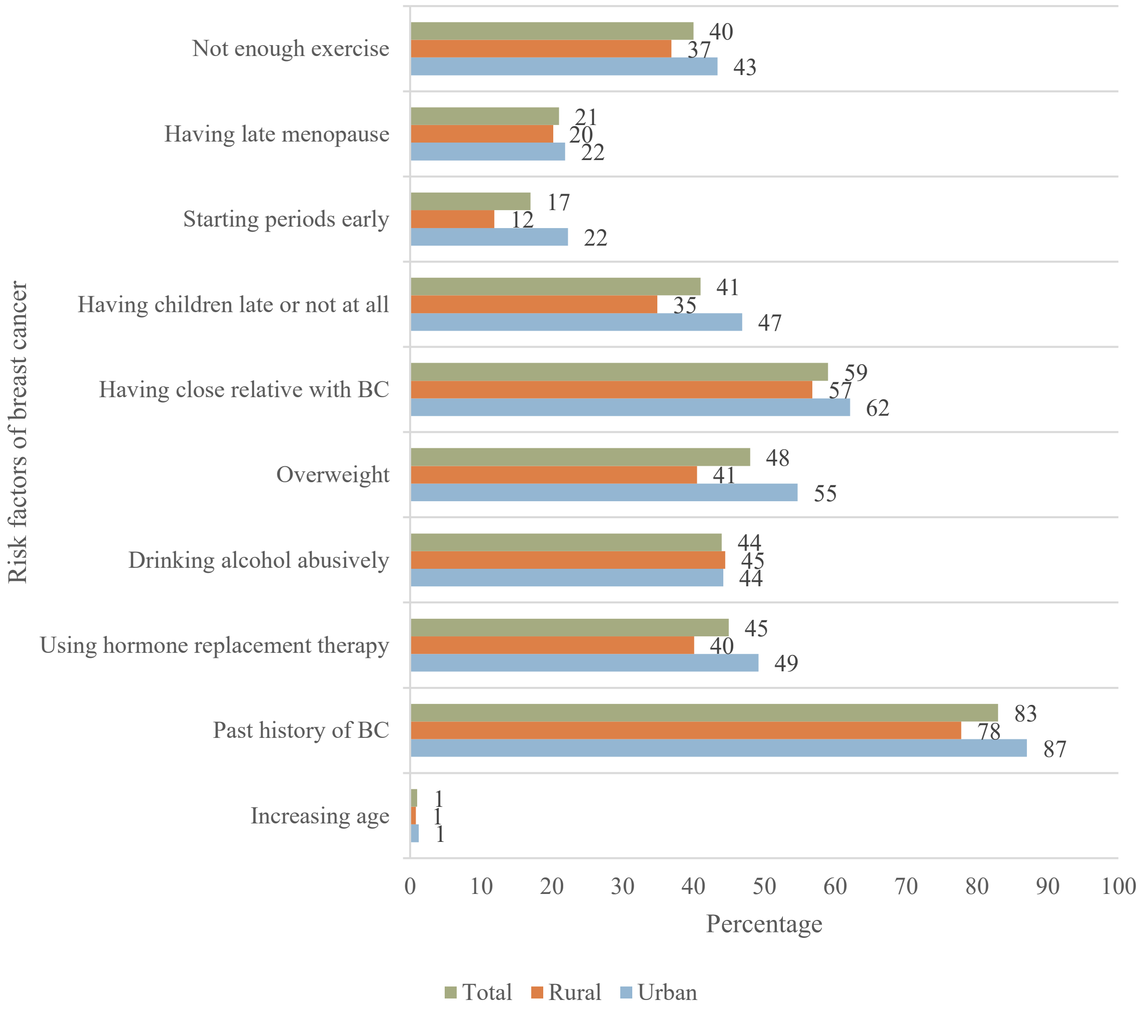

S3 Fig. Proportion of respondents who could correctly identify screening modalities of breast cancer, by residence area.

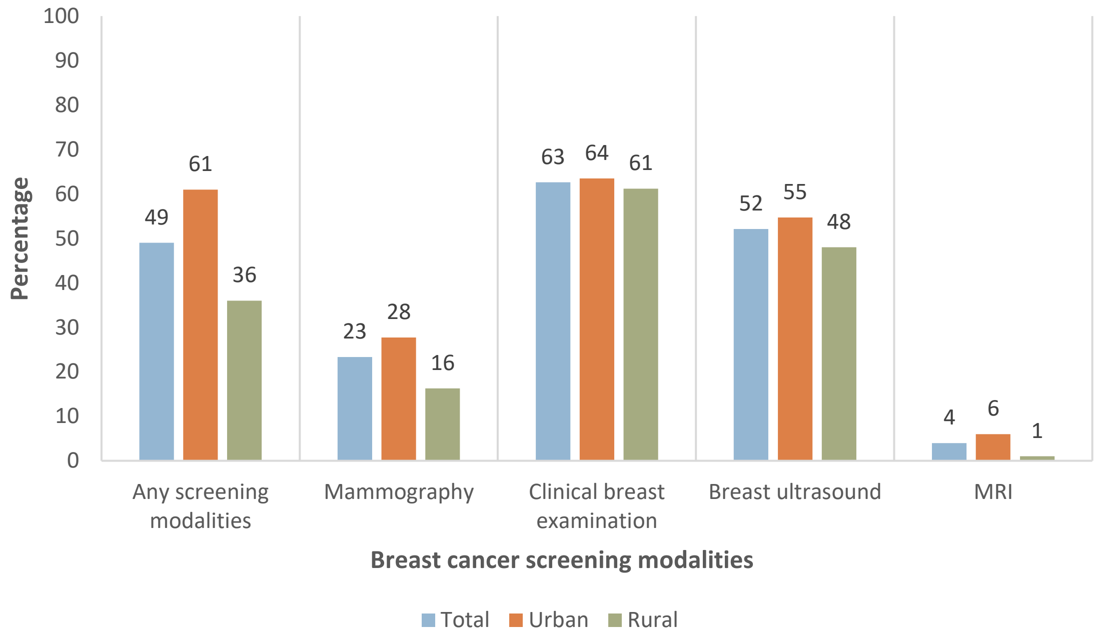

1. Vietnam General Statistics Office, Central Population and Housing Census Steering Committee. Vietnam Population and Housing Census 2009. Hanoi, Vietnam: Statistical Publishing House; 2010.
