# Supplementary material for: Breast cancer screening practices among Vietnamese women and factors associated with clinical breast examination uptake
Source: PLoS One. 2022 May 27;17(5):e0269228. doi: 10.1371/journal.pone.0269228 (PMC9140272; doi:10.1371/journal.pone.0269228)
Supplement: Attachment — Submitted filename: Response to reviewers.docx [file pone.0269228.s003.docx]

**RESPONSE TO REVIEWERS’ COMMENTS**

**REVIEWER 1**

The subject matter of this manuscript is a global public health issue and very critical for all countries. The authors have presented the results in an intelligible fashion in mostly simple and clear language. The sample size is large and seem representative for the Vietnamese women of reproductive age. The results are well analysed and presented clearly and the statistical analysis are robust. The discussion section is also presented concisely, and the conclusions are drawn based on the results presented.

I do have a few suggestions that I think will make the manuscript even more meaningful to all readers.

1. Please, provide more elaboration on the ethical review and approval process in addition to the approval number and the statement that participants gave consent prior to be being included in the study. What kind of consent was given? Written or verbal and how was participants informed and convinced to participate and to consent?

***Response:*** Thank you for your feedback. We have added more details about the type of informed consent and the procedure that was used to obtain consent. The revised paragraph is as follows:

“All respondents received an information sheet about the study including information about its voluntary nature, objectives, target respondents, privacy, use of collected data, potential drawbacks, and benefits of participation. Interviewers also provided respondents with a verbal explanation about the study’s purpose and the interview procedure. Written informed consent was obtained from all respondents who agreed to participate.” (Line 91-96)

2. Please, provide some explanation on the inclusion and exclusion criteria for the study besides the age limitation.

***Response:*** The inclusion criteria were (i) women aged 30-74 years, (ii) never had breast cancer (self-reported), and (iii) consented to participate in the survey. These were mentioned in the methods section, line 82-83.

3. In the results presentation, you used statements such as "approximately, less than, about around two-thirds". Please, be straight forward and use the exact percentages instead of the probable phrases before the percentages in parenthesis.

***Response:*** Thank you for your feedback. We have revised the whole results section and we have used the exact percentages as suggested.

4. Please, be consistent with the use of either Vietnam women or Vietnamese women instead of using them interchangeably. However, I could be wrong as both could be acceptable in Vietnam. Please, advise.

***Response:*** Thank you for your feedback. It should be Vietnamese women. We have fixed the typo and ensured that the right term is used throughout the paper.

5. There are a few vocabulary issues, and a careful edit will resolve them, please.

***Response:*** Thank you for your feedback. Our English-speaking co-authors have carefully edited the paper.

6. In the discussion section, please, provide more relevant studies in the literature related to your study and do the relevant comparisons.

***Response:*** Thank you for your feedback. We have provided 15 studies including studies from Vietnam, China, Hong Kong, Malaysia, and the United Kingdom (the birthplace of the Breast-CAM instrument) and compared the results of these studies with the results of our study. We were careful to include consideration of studies from countries that were relatively close in culture with Vietnam; thus, our assessment was that comparisons with studies in the Americas, Africa, Europe, and Australia were of limited comparative value.

7. Please, add a section on strengths and limitations and add the statement on your use of standardized instrument compared to personally developed instruments as a strength. Then provide a weakness if there a challenge you encountered.

***Response:*** Thank you for your feedback. The last paragraph of the discussion section is dedicated to discussing the strengths and limitations of the study. In particular, it mentions that “This is the first study in Vietnam to use standardised instruments (i.e., Breast-CAM and CHBMS) to assess BC knowledge, attitudes, and beliefs among Vietnamese women. As such, it allows comparisons to be made with studies from other countries and provides reliable and valid data that contributes to improved understanding about BC knowledge and awareness and aids the planning of BC interventions and related policy in Vietnam.”

We have added more signposting to this key paragraph, for example, “The study has certain strengths”, and “Apart from these strengths, the study also has some limitations”.

**REVIEWER 2**

Given the absence of a copy editor, it would serve the authors well to have the manuscript proof-read and edited to improve conciseness.

***Response:*** Thank you for your feedback. Our English-speaking co-authors have carefully edited the paper.

Abstract:

Was the instrument cross culturally adapted, translated, back translated? Please elaborate.

***Response:*** Thank you for your feedback. The instruments (Breast-CAM and CHBSM) were translated into Vietnamese by the first author who is Vietnamese and bilingual. Back translation was carried on by another Vietnamese researcher working at the Centre for Population Heath Sciences – Hanoi University of Public Health. We compared and discussed the two versions and made some minor amendments. This revised Vietnamese version of the questionnaire was piloted by interviewing 10 volunteer respondents in Hanoi. The results of the pilot interviews were used to finalise the questionnaire used in the main data collection exercise. We have added the above information to the manuscript (Line 128-133) but could only squeeze a reference to this point in the abstract due to words restriction.

Was IRB approval obtained, were patients consented. Based on answers to questions, it appears they did. But might be better to state this in methods: In this IRB-approved study, consented patients responded to a 300-item scale cross culturally adapted from the …”

***Response:*** Thank you for your feedback. We provided the following information in the methods section, “The study received ethical approval (ref no: 319/2019/YTCC-HD3, dated 30th May 2019) from the Hanoi University of Public Health’s Institutional Review Board. Informed consent was obtained from all respondents who agreed to participate” (Line 90-91, 95-96). Based on recommendations from the editorial board and the other reviewer, we have added information about the type of consent (written) and the procedure that was used to obtain consent from respondents - “All respondents received an information sheet about the study including information about its voluntary nature, objectives, target respondents, privacy, use of collected data, potential drawbacks, and benefits of participation. Interviewers also provided respondents with a verbal explanation about the study’s purpose and the interview procedure.” (Line 91-95).

We have made a reference only to these details in the abstract due to words restriction.

When you say, “18% of V. Women had knowledge’” what does that mean – this percentage discussed with someone, heard about breast cancer, …

***Response:*** Thank you for your feedback. This statement means that 18% of women were aware of the symptoms, risk factors, and screening modalities for BC - Questions were posed about three categories of knowledge: ‘BC symptoms’, ‘BC risk factors’, and ‘BC screening modalities’. Definition of ‘having knowledge’ in each component was provided in the methods section (be able to identify more than five non-lump symptoms out of 9; identify more than five risk factors out of 10; and name at least one correct screening modality, respectively) (line 152-159). Women who had knowledge in all three components were defined as ‘having knowledge of’, or ‘being knowledgeable about BC.” (Line 153, 154). We cannot add these details to the abstract due to the word count restriction.

Introduction:

Excellent first paragraph with high yield information. It might help to mention that in the absence of a population-based screening program, early detection (of which awareness is key) is essential.

***Response:*** Thank you for your feedback. We mentioned “Although early detection through screening is critical in this context, Vietnam currently does not have a national BC screening programme.” (Line 54-55)

Second paragraph:

The second sentence is inaccurate and should be removed. In fact, mammography is highly useful in this age group. It does have reduced sensitivity in dense breast women, which is more common in Vietnamese and young women for a variety of reasons. However, it is still effective. Also, there are multiple reasons to list to describe why mammography is not a good option in LMICs.

Unlike first paragraph, where authors provided specific data to support their research, the second paragraph lacks data and speaks generally. I would prefer more information here. I think a lot of this paragraph is unnecessary and could be more concise to present data without increasing word count.

***Response:*** Thank you for your feedback. We have revised the second paragraph (lines 56-65) to take account of these points and to provide a clearer and more concise message. In addition, the paragraph emphasises the importance of acceptability to the goal of achieving successful implementation and uptake of screening. A programme may be cost-effective for a LMIC but may fail to impact if acceptability and uptake is low as, for example, the experience in the Philippines.

Methods:

Paragraph 1:

Please state why you chose neighbourhoods in Hanoi and what you did when someone wasn't home (did you return, leave your number). What days and times of days did you visit? (If you visited during weekday, then maybe women at home were more likely new mothers or unemployed, or…). It helps reader understand bias that might be present/how generalizable the results are.

***Response:*** Thank you for your feedback. Hanoi was chosen for pragmatic reasons: it was the second most populous city/province in Vietnam and, so, data collection could be undertaken within the limited resources of the study. Also, Hanoi has an almost equal number of urban and rural districts (13 urban districts and 17 rural districts) - a characteristic that is hard to find in other provinces. This feature ensured that we were able to draw a sample that comprised women from urban and rural communities.

Data collection was carried out on August 17^th^ and 18^th^, 2019 (a weekend) in the rural district and between August 23^rd^ and 26^th^, 2019 (24^th^ and 25^th^ were weekend days) in the urban district. In each district, two supervised teams of five interviewers conducted interviews simultaneously in two communes selected using Probability proportional to size (PPS) sampling. Data collection lasted longer in the urban area as the teams could interview women only early in the morning or late afternoon instead of throughout the whole day as was possible in the rural area - the proportion of urban dwelling women who worked as full-time employees was higher. We drew upon the female age structure and other socio-demographic information that was reported in the Vietnam Census 2009 to ensure that the sample was representative proportionally of each age group and did not comprise only old women and other social variables (e.g., unemployed).

We have added these details to the methods section, line 140-145.

I think the ethical statement should be at front of paragraph, but this is more stylistic.

***Response:*** Thank you for your feedback.

Was the instrument translated, back translated, who translated? Were the interviewers trained, what experience did they have?

***Response:*** Thank you for your feedback. The instruments (Breast-CAM and CHBSM) were translated into Vietnamese by the first author who is Vietnamese and bilingual. Back translation was carried on by another Vietnamese researcher working at the Centre for Population Heath Sciences – Hanoi University of Public Health. We compared and discussed the two versions and made some minor amendments. This revised Vietnamese version of the questionnaire was piloted by interviewing 10 volunteer respondents in Hanoi. The results of the pilot interviews were used to finalise the questionnaire used in the main data collection exercise.

10 interviewers were recruited from the Hanoi University of Public Health’s 3rd and 4th year student cohorts who had experience of working as community survey interviewers. Two research assistants at Hanoi University of Public Health acted as field supervisors and assisted the interviewers. Both interviewers and field supervisors received a 1-day-training course prior to data collection. The training included an introduction to the study objectives, how to select households and respondents, questionnaire briefing, and a practice session.

We have added the above information into the manuscript (Line 128-133 and 135-139).

Results:

It would help to see the instrument. I’m not sure what a “non-lump symptom” is or what the authors considered risk factors.

***Response:*** Thank you for your feedback. The Breast-CAM instrument is accessible and free to download from the website of its developer – Cancer Research UK. The URL is <https://www.cancerresearchuk.org/health-professional/awareness-and-prevention/the-cancer-awareness-measures-cam>. We have added a footnote with this link for readers who would like more details about instrument content.

The instrument poses questions/items about 11 symptoms and 10 risk factors. The list of symptoms and risk factors along with the proportion of respondents who correctly identified each item are provided as Supplementary material (S1 Fig and S2 Fig).

Pain is a late symptom. In fact, studies have shown that pain alone as a symptom is unlikely to be related to breast cancer. Is this what you want women, particularly young women, to learn. Part of raising awareness is to limit overburdening the healthcare system and teaching “pain” as a breast cancer symptom could have opposite effect.

***Response:*** Thank you for your feedback. We agree that pain in and around the breasts/armpits may relate to diseases/conditions other than breast cancer. However, the Breast-CAM is a proven valid and reliable instrument for measuring breast cancer awareness and, so, pain may be a sign of breast cancer, along with 10 other symptoms that are listed in the questionnaire. Knowledge about symptoms was evaluated based on the answers to all 11 symptoms. The Breast-CAM is a way to quantifying the nature and level of knowledge about BC among women.

Breast cancer knowledge is presented as a scale. Did they check the relationship between the items?

***Response:*** Thank you for your question. Breast cancer knowledge is not a continuous variable (scale) but a dichotomous variable with two values ‘yes’ (having knowledge) or ‘no’. On the other hand, the CHBMS (a measure of beliefs or perceptions about BC and BC screening) has been used as a scale. We have calculated the relationship between the items in the CHBMS and presented the result in the form of Cronbach’s α which ranged from 0.75 to 0.88 in the original study^[1]^ and from 0.69 to 0.78 in this study (Line 126, 127).

Discussion:

Please use first paragraph to state the most important finding of the study and what implication this has on the Vietnamese population (as it pertains to breast cancer)

***Response:*** Thank you for the suggestion. We have restructured and revised the whole discussion section. As suggested, we have added a first paragraph (line 239-244) to report the uptake of CBE and its associated factors as well as the implication of these findings (high acceptance towards CBE and feasibility of implementation).

Authors do a good job of stating their results and stating (usually) how this relates to Vietnamese population, but then fail to drive home the “so what”. Also, the discussion lacks conciseness and synthesis of the results. I would avoid showing data here unless it helps convey a point you make in the paragraph.

***Response:*** Thank you for your feedback. We have revised the whole discussion section to make it more concise. Data is not shown unless necessary (for example when comparing with other studies) as suggested. Key messages and implications are presented in the first paragraph of the discussion section (line 239-244) and in the conclusion section. For example, “Current engagement and the absence of socio-demographic disparities indicate that a CBE programme is likely to produce positive outcomes for women in Vietnam and their families and wider society” (line 314-316).

1. Champion VL. Revised susceptibility, benefits, and barriers scale for mammography screening. Research in nursing & health. 1999;22(4):341-8.
