# Supplementary material for: Breast cancer screening practices among Vietnamese women and factors associated with clinical breast examination uptake
Source: PLoS One. 2022 May 27;17(5):e0269228. doi: 10.1371/journal.pone.0269228 (PMC9140272; doi:10.1371/journal.pone.0269228)
Supplement: Attachment — Submitted filename: Response to reviewers.docx [file pone.0269228.s004.docx]

**RESPONSE TO REVIEWERS’ COMMENTS**

**REVIEWER 1**

This is a much-improved version and a very sound and important research. In order to improve the manuscript even better and increased the possibility of its acceptance, I suggest the following.

1. Please, all your tables need to be appropriately formatted. Look up examples of APA table format and use it. It is much better presented than your current tables.

***Response:*** Thank you for your feedback. We understand the APA table format limits the use of borders or lines in a table. However, the formatting requirements from Plos One are different in the way that all borders and lines must be shown^[1]^.

2. You need to provide subheadings for all the variables or analysis in the findings section. These include knowledge of BC, Health Model, CBE screening, and logistic regression model results.

***Response:*** Thank you for your feedback. We have added the subheadings as recommended.

3. In the findings section line 225, you indicated F1 but there no figure and there is no description. Please, delete or clarify

***Response:*** Thank you for your feedback. As formatting instruction for manuscript submitting to Plos One, Figure 1 caption was inserted in the text of the manuscript, immediately following the paragraph in which the figure is first cited (line 226). The figure was submitted as an individual file. We are sorry for the confusion but we followed the requirements of the journal.

4. Using i.e should be avoided. Instead, write it in full

***Response:*** Thank you for your feedback. The use of i.e., is common in English formal writing and does not against the journal’s formatting requirements. i.e.," stands for Latin "id est"; thus, we think it is more confusing if write in full the original Latin words.

5. You need to be consistent with spelling regimes. Use programme throughout or program but do not in a single manuscript.

***Response:*** Thank you for your feedback. We have fixed the typo and ensured the word ‘programme’ was used throughout in the manuscript.

6. On line 290, use the term differentiated public health education programme. A more appropriate terminology is targeted public health where populations are divided into segments depending on some of criteria.

***Response:*** Thank you for your feedback. We have added changed the terminology to the suggested one.

**REVIEWER 2**

The authors wrote a substantially improved version of their prior manuscript.

Small stylistic suggestion would include writing in the active voice. For example, rather than writing, "Instead, clinical breast examination (CBE) – an alternative low-cost screening tool with downstaging effect – is more likely to represent a realistic intervention in LMICs [8] and it has been shown to be cost-effective in Vietnam [9].", I suggest writing, "In contrast, clinical breast Examination (CBE) represents...".

***Response:*** Thank you for your feedback. We have amended the writing and used active voice wherever possible.

Second sentence of discussion: Consider specifying which types of screening (All types/modalities?) you include in this percentage. For example, "Among all types of breast cancer screening,..." or "More than 63% of participants reported participating..."

***Response:*** Thank you for your feedback. We have specified the types of screening mentioned in the sentence ‘among all types of breast cancer screening…’, ‘63% of respondents reported participating in at least one type of BC screening modality’.

1. <https://journals.plos.org/plosone/s/tables>
